# Cyclooxygenase 2 Effector Genes as Potential Inflammation-Related Biomarkers for Colorectal Cancer Circulating Tumor Cells Detection by Liquid Biopsy

**DOI:** 10.3389/fphar.2021.806395

**Published:** 2022-01-28

**Authors:** Konstantinos Stamatakis, Patricia Torres-Gérica, Alba Jiménez-Segovia, Edurne Ramos-Muñoz, Lorena Crespo-Toro, Patricia Fuentes, María L. Toribio, Francisco Callejas-Hernández, Alfredo Carrato, María Laura García Bermejo, Manuel Fresno

**Affiliations:** ^1^ Centro de Biología Molecular Severo Ochoa, UAM/CSIC, Madrid, Spain; ^2^ Department of Molecular Biology, Universidad Autónoma de Madrid, Madrid, Spain; ^3^ Instituto Ramón y Cajal de Investigación Sanitaria, Madrid, Spain

**Keywords:** colorectal cancer, liquid biopsy, circulating tumor cells, cyclooxygenase 2-regulated genes, Parsortix system, inflammation-related genes

## Abstract

Cyclooxygenase 2 (COX2) has been implicated in cancer development and metastasis. We have identified several COX2-regulated inflammation-related genes in human colorectal cancer cells and shown that some of them play important roles in tumor progression. In this work, we have studied the COX2-regulated genes in the mouse colorectal cancer cell line CT26, to find that many are also regulated by COX2 over-expression. On the other hand, we generated a CT26 cell line expressing Gfp and Luciferase, to study tumor growth and metastasis in immunocompetent Balb/c mice. We then collected solid tissue, and blood samples, from healthy and tumor-bearing mice. Using the Parsortix^®^ cell separation system and taking advantage of the fact that the tumor cells expressed Gfp, we were able to identify circulating tumor cells (CTCs) in some of the mice. We compared the mRNA expression levels of Ptgs2 and effector genes in the samples obtained from tumor-bearing or healthy mice, namely, tumor or healthy colon, Ficoll purified buffy coat, and Parsortix-isolated cells to find different patterns between healthy, tumor-bearing mice with or without CTCs. Although for genes like Il15 we did not observe any difference between healthy and tumor-bearing mice in Ficoll or Parsortix samples; others, such as Egr1, Zc3h12a, Klf4, or Nfat5, allowed distinguishing for cancer or CTC presence. Gene expression analysis in Ficoll or Parsortix processed samples, after liquid biopsy, may offer valuable diagnostic and prognostic information and thus should be further studied.

## Introduction

Colorectal cancer (CRC) is the second cause of cancer death in the developed countries. In the last decade, a small decrease in the death numbers caused by this type of cancer has been achieved, mainly due to prevention and early screening (Siegel et al., 2020). The recent COVID-19 outbreak has led to a drastic reduction (86%) of early screening (Patel et al., 2020), which leads to the increase of advanced cancer cases (Aguiar et al., 2021) and the need to implement new prognostic tools and treatment strategies. Circulating tumor cell (CTC) enumeration is used as a prognostic tool in different cancer types, among them, metastatic colorectal cancer (Sastre et al., 2012; Sotelo et al., 2015). The CTCs have been found to be very heterogenous, varying in surface marker expression, which reduces the value of FDA-approved tools, such as the CellSearch system (Gervasoni et al., 2011; Raimondi et al., 2014). Taking advantage of the physical characteristics of the CTCs, rather than the specific surface or expression markers, to isolate them, the Parsortix system has been able to isolate CTCs from a great variety of tumors and patients and it has been used also in preclinical models (Kitz et al., 2018; Obermayr et al., 2018; Obermayr et al., 2019).

Cyclooxygenase 2 (COX-2) is widely accepted to be implicated in CRC progression and metastasis, as well as an important therapeutic target for treatment or prevention of this type of cancer (Doherty and Murray, 2009; Wang and DuBois, 2010). In an effort to identify the mechanisms through which COX-2 activity leads to CRC progression, we showed that its overexpression is enough to increase cancer cell aggressiveness (Stamatakis et al., 2015) through the regulation of the expression of genes we consider COX-2 activity effectors in the cancer cells (Hidalgo-Estévez et al., 2020). A recent study pointed out that COX-2 is expressed in CTCs of CRC patients and associated with the clinicopathological features of the patients (Cai et al., 2019).

We decided to study the effect of COX-2 and its effector genes in a mouse model of colorectal cancer and metastasis. We confirmed that COX-2 activity had similar effects on the COX-2-effector genes as in human CRC cells. Moreover, we studied the expression of these genes, as well as *Ptgs2*, the mouse gene encoding Cox-2, in tumors, peripheral blood-nucleated cell isolates, and in CTCs isolated with the Parsortix system. We found that the expression levels of this group of genes can help estimate the presence of CTCs and that this strategy could be useful to identify new molecular markers for CTCs.

## Materials and Methods

### Mice and Mouse Models

All animal studies were done according to Spanish and European regulations, the Ethics Committee of Animal Experimentation (CSIC-UAM), and the Institutional Review Board of UAM. Six-week-old female Swiss *Nude* and Balb/c mice were obtained from Janvier labs. For subcutaneous inoculation, 10^6^ cells were injected under the skin of the left flank of the mice. Orthotopic inoculation in the cecum wall of 50,000 cells was done according to the method described by Tseng et al. (2007). Tumor growth was monitored by bioluminescence, using an IVIS Lumina system (Perkin Elmer), as described before (Jiménez-Segovia et al., 2019). Fifteen mice were inoculated, 11 of which developed tumors. At the end of the experiment, mice were sacrificed by CO_2_ inhalation, blood was collected through heart puncture, and tumors were excised for further processing (macroscopic separation of tumors and RNA extraction). From the 11 blood samples, four were discarded due to extensive clotting (although all animals were injected intraperitoneally with heparin to avoid this). Samples from five healthy mice were used as controls.

### Cell Culture

Colorectal carcinoma cell line CT26 was obtained from the ATCC and maintained according to the distributor’s instructions. Human COX2 overexpression was achieved after transduction with a lentiviral vector and antibiotic selection, as described before Stamatakis et al. (2015). The CT26-Luc-Gfp cell line was generated by transducing CT26 cells with lentiviral particles generated with the pHRSIN-Luc-IRES-Gfp vector, originally described in Garaulet et al. (2013), carrying the firefly luciferase and Gfp cDNAs separated by an IRES. Gfp-positive cells were sorted with a FACSAria Fusion (BD Biosciences), and light-emitting clones were selected, adding D-luciferin to the medium and detecting light emission with a BMG Biosciences FluoStar Optima plate reader. After inoculation and tumor growth in *nude* mice, tumors were excised and tumor cells cultured and sorted for Gfp positive cells. These cells were used for further experiments in Balb/c mice.

### Reagents and Materials

All reagents were purchased from Sigma Aldrich, unless stated otherwise. Oligonucleotides were synthetized by Sigma Aldrich, according to the following sequences (gene symbol, forward primer, reverse primer): *Ptgs2*, 5′ gat​gct​ctt​ccg​agc​tgt​gc, 5′ gga​ttg​gac​agc​aac​cat​ttg; *Ptges*, 5′ gtg​atc​tcc​tgg​ctg​caa​atc, 5′ cct​gga​cag​tgc​ttt​gct​ctg; *Dusp10*, 5′ cct​gtc​gtc​taa​agg​aga​tgg​a, 5′ cag​atg​gta​gag​ggc​tcg​c; *PMEPA1*, 5′ gac​cat​ctt​cga​cag​tga​cct, gta​gca​ggt​ggc​gct​gat​g; *KLF4*, 5′ atg​gtc​aag​ttc​cca​gca​ag, 5′ ttt​ctg​ttt​tgt​ctc​ttg​aac​tct​tc; *Tacstd2*: 5′ cgg​gca​aat​aca​aaa​agg​tg, 5′ aca​agc​tag​gtt​cgc​ttc​tca; *Zc3h12a*, 5′ tca​tcg​acg​gaa​gca​atg​t, 5′ cct​cgc​tcc​aga​aac​cag; *Nfat5*, 5′ tca​gac​aag​cgg​tgg​tga, 5′ agg​gag​ctg​aag​aag​cat​ca; *Ptgfr*, 5′ tgc​aat​gtt​ggc​cat​tgt​tac​g, 5′ ctg​gcc​ata​atg​tgc​gtc​tc; *Egr1*, 5′ tca​cct​ata​ctg​gcc​gct​tc, 5′ ggt​tca​ggc​cac​aaa​gtg​ttg; *Tgfb1*, 5′ cca​agg​taa​cgc​cag​gaa​ttg​ttg​cta​ta, 5′ agc​gga​cta​cta​tgc​taa​aga​ggt​caa​cc; *Inhba1,* 5′ tcc​tct​tca​tgg​tat​tgg​ca, 5′ ggg​agt​gat​ccc​tgg​aaa​c; *Il15ra,* 5′ tgc​aga​agt​tgt​ttg​gga​tg, 5′ tac​ccg​caa​tga​cca​cag​aga; *Il15*, 5′ act​gtc​agt​gta​taa​agt​ggt​gtc​aat​atg, 5′ cag​agg​cca​act​gga​tag​atg​taa​g; *Nfkbia*, 5′ cga​gga​gta​cga​gca​aat​gg, 5′ tga​ttg​cca​agt​gca​gga; *Gadph*, 5′ tgt​aga​cca​tgt​agt​tga​ggt​ca, 5′agg​tcg​gtg​tga​acg​gat​ttg.

### Isolation of Blood-Nucleated Cells (Ficoll Samples)

Mouse blood was collected in EDTA-treated tubes, to avoid coagulation; 1 ml of Ficoll Paque (FisherSci) was placed beneath the blood in the tube and centrifuged at 1,500 rpm with for 30 min according to the manufacturer´s instructions. The upper, clear phase containing all nucleated cells was collected, diluted in PBS, and centrifuged to obtain the cell pellet.

### Isolation of CTCs With the Parsortix System

Mouse blood was isolated as above, diluted 1:1 with PBS, and introduced to the Parsortix system (Angle PLC, Surray, United Kingdom), according to the manufacturer´s instructions, collecting the CTCs in a 6.5 μm cassette using the Parsortix PX2_S99F protocol. Once the protocol was finished, the cassette was removed and observed under an Axiovert200 (Zeiss) fluorescence microscope with a sCMOS monochrome camera for presence of Gfp-positive cells (CTCs, since only tumor cells are Gfp-positive). To avoid cell loss or RNA degradation, cells were immediately lysed in the cassette through the flow of 300 μl of isolation buffer (SPLIT RNA extraction kit), collecting all the flowthrough volume, before proceeding to RNA extraction. Parsortix system-isolated CTCs have been characterized in other studies (Gkountela et al., 2019; Szczerba et al., 2019).

### Gene Expression Analysis

RNA was extracted using Trizol reagent (Thermo Fisher Scientific) for cell culture or tumor samples and with the SPLIT RNA extraction kit (Lexogen) for Parsortix cassette and Ficoll samples. The RNA was retrotranscribed with the Transcriptor First Strand cDNA Synthesis Kit (Roche Diagnostics). Quantitative PCR was performed using the GoTaq 2-Step RT-PCR system (Promega). Relative mRNA levels to the *Gadph* housekeeping gene (ΔCt) and to the experimental control point (ΔΔCt) were calculated using the 2^−ΔΔCT^ formula from the values obtained. To quantify and compare gene mRNA levels between Parsortix samples (less than 1,000 cells), Ficoll-isolated buffy coats (millions of cells), and tumor samples (millions of cells), the entire eluate of the SPLIT RNA extraction kit was used for the first type of samples, while 50 ng RNA was used for the rest, for retrotranscription. Pre-amplification and qPCR amplification were performed using the RealTime ready cDNA Pre-Amp Master (Roche Diagnostics) with the RealTime ready Pre-Amp Primer Pool, and the RealTime ready custom panel (Roche Diagnostics) according to the manufacturer´s instructions, using a LightCycler 480 (Roche Diagnostics). mRNA levels of each gene were expressed as ΔCt, the difference of the crossing point (Ct) of the gene with the Ct of the housekeeping *Gadph*.

### Statistical Analysis and Principal Component Analysis

Statistical analysis was performed with the Graphpad Prism software. Depending on the data compared, unpaired *t*-test, Wilcoxon test, or Mann–Whitney *U* test was performed. Significance is indicated in the figure legends.

Principal component analysis (PCA) (Mardia et al., 1979; Becker et al., 1988; Venables and Ripley, 2002) was conducted in RStudio 2021.9.0.351 (R Core Team, 2021 https://www.rstudio.com/) using *prcomp* command, and *ggbiplot* package (Vincent Q. Vu 2011 https://github.com/vqv/ggbiplot) was used for data visualization.

## Results

### COX2-Regulated Genes in CT26 Cells

**FIGURE 1:**
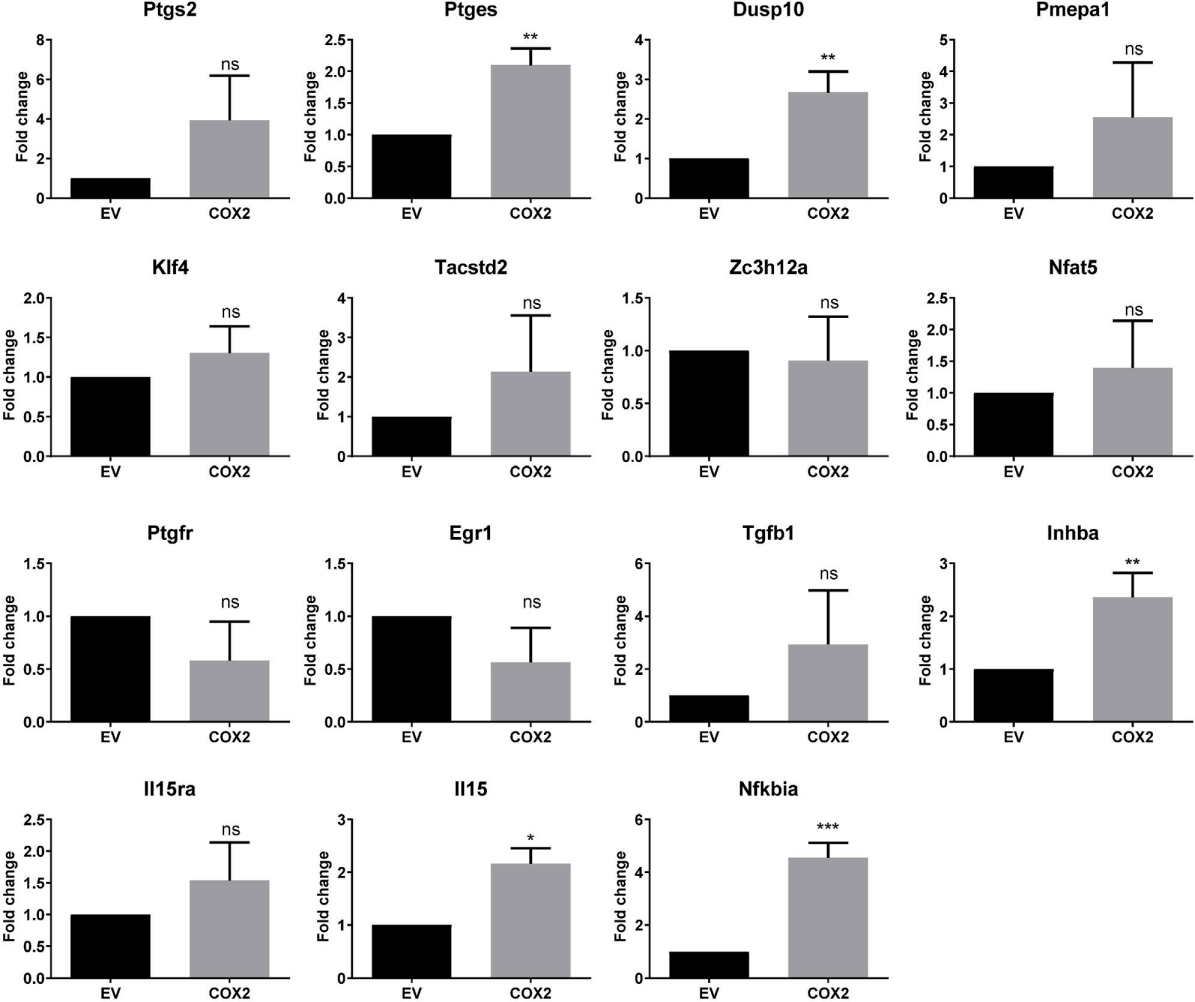
COX2-regulated gene expression in CT29 cells. Fold change of mRNA levels in CT29- COX2 cells compared to the empty vector ones (EV) as estimated by RT-qPCR. Statistically significant differences were tested using the unpaired *t* test. ns: not significant; *: *p* < 0.05; **: *p* < 0.001; ***: *p* < 0.0001.

**FIGURE 2:**
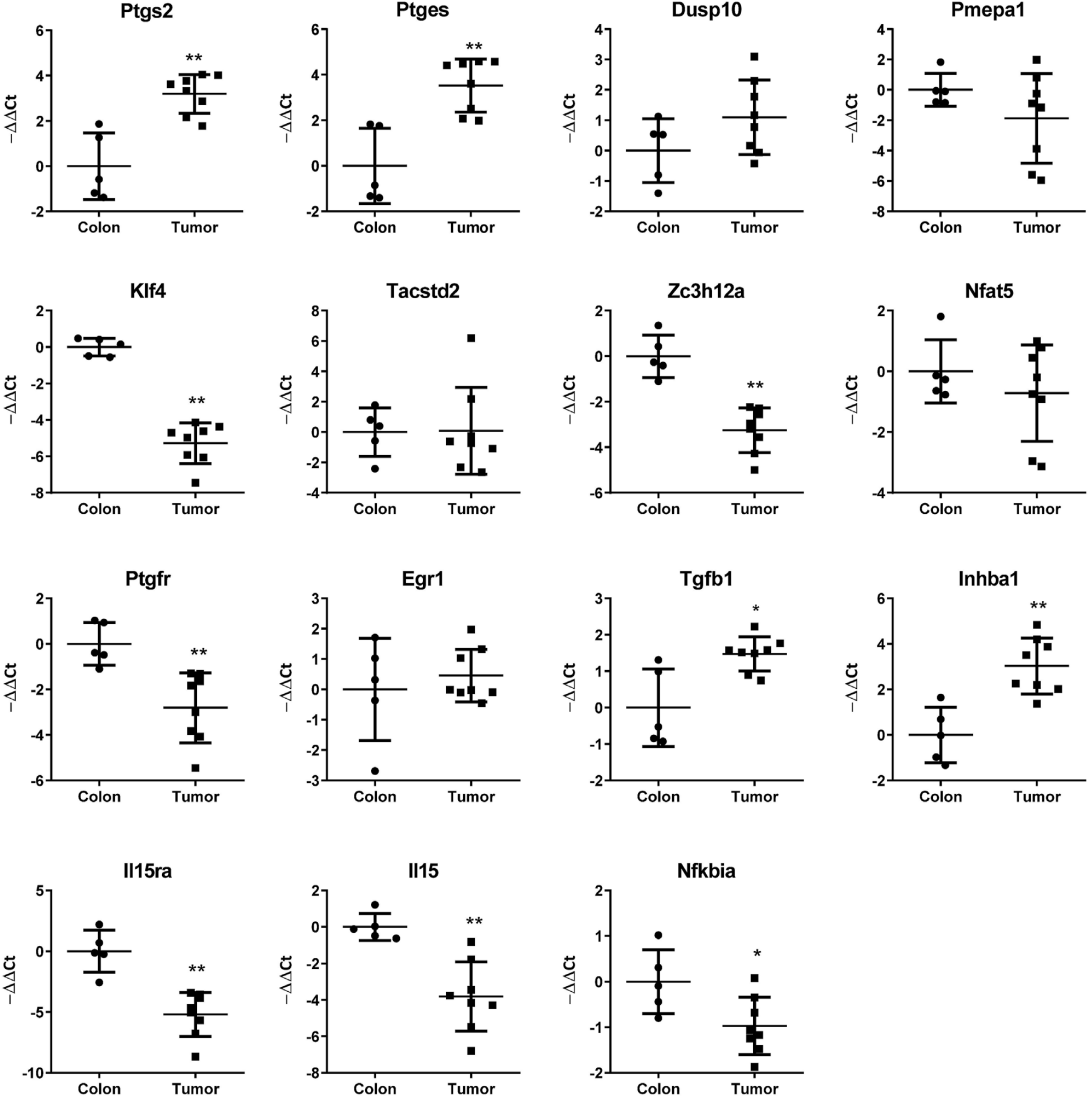
mRNA levels of the indicated genes in mouse colon and CT29 cell-derived orthotopic allografts as estimated by RT-qPCR. −ΔΔCT values are shown (ΔCTnormal colon-ΔCTsample). Mann–Whitney *U* test was performed for statistical significance of the differences in gene expression. *: *p* < 0.05; **: *p* < 0.001.

We have identified in previous works (Stamatakis et al., 2015; Hidalgo-Estévez et al., 2020) genes regulated by cyclooxygenase activity in HT29 human colon cancer cells. Among the upregulated genes were *PTGES*, *DUSP10*, *PMEPA1*, *KLF4*, *TACSTD2*, *ZC3H12A*, *NFAT5*, *PTGFR*, *EGR1*, *TGFB1*, *INHBA*, *IL15RA*, *IL15*, and *NFKBIA*. In this work, we overexpressed COX2 in the mouse colon cancer cell line CT26, to test if these genes were also upregulated. CT26 cells in culture express low levels of *Ptgs2*, which increased when we overexpressed the human COX2, probably through a positive feedback loop. Although there was a tendency for many of the mouse homologs of the mentioned genes to be upregulated, we found that this was significant only for *Ptges*, *Dusp10*, *Inhba*, *Il15*, and *Nfkbia* (Figure 1). COX2 overexpression has been associated with colorectal cancer progression and metastasis, it is upregulated in tumors vs. normal colon tissue and we have shown that *PTGS2* (that encodes COX2) is also up-regulated in human xenografts in nude mice (Stamatakis et al., 2015). Using the CT26-Balb/c syngeneic, orthotopic mouse model, we tested if the mRNA levels of *PTGS2* and the abovementioned effector genes varied between normal colon and tumor tissue. As it can be seen in Figure 2, *Ptgs2* levels are significantly higher in tumor tissue than normal colon. Similar results were obtained for *Ptges*, *Dusp10*, and *Inhba1*, suggesting that the *Ptgs2* upregulation could be responsible for their increased mRNA levels. On the contrary, *Pmepa1*, *Klf4*, *Il15ra*, *Il15*, and *Nfkbia* were significantly decreased indicating that other factors may be more important than the cyclooxygenase activity for their regulation *in vivo*. We also checked *Ptgfr* expression levels to find that both in culture as *in vivo*, it had an inverse correlation with Ptgs2 expression levels. Moreover, when comparing data from our mouse model and from the TCGA colon cancer cohort analyzed using the UCSC Xena browser (https://xenabrowser.net/), the direction in the gene expression change, between normal tissue and tumor, was similar in both human and mouse for *Ptgs2*, *Ptges*, *Dusp10*, *Klf4*, *Ptgfr*, *Egr1*, *Inhba1*, *Il15*, and *Nfkbia* (not shown). The rest of the genes had a different behavior when comparing colon and tumor tissue, suggesting there are differences in gene regulation and in their role in cancer progression between the two species.

### The CT26-Balb/C Orthotopic Colon Cancer Model Produces CTCs

**FIGURE 3:**
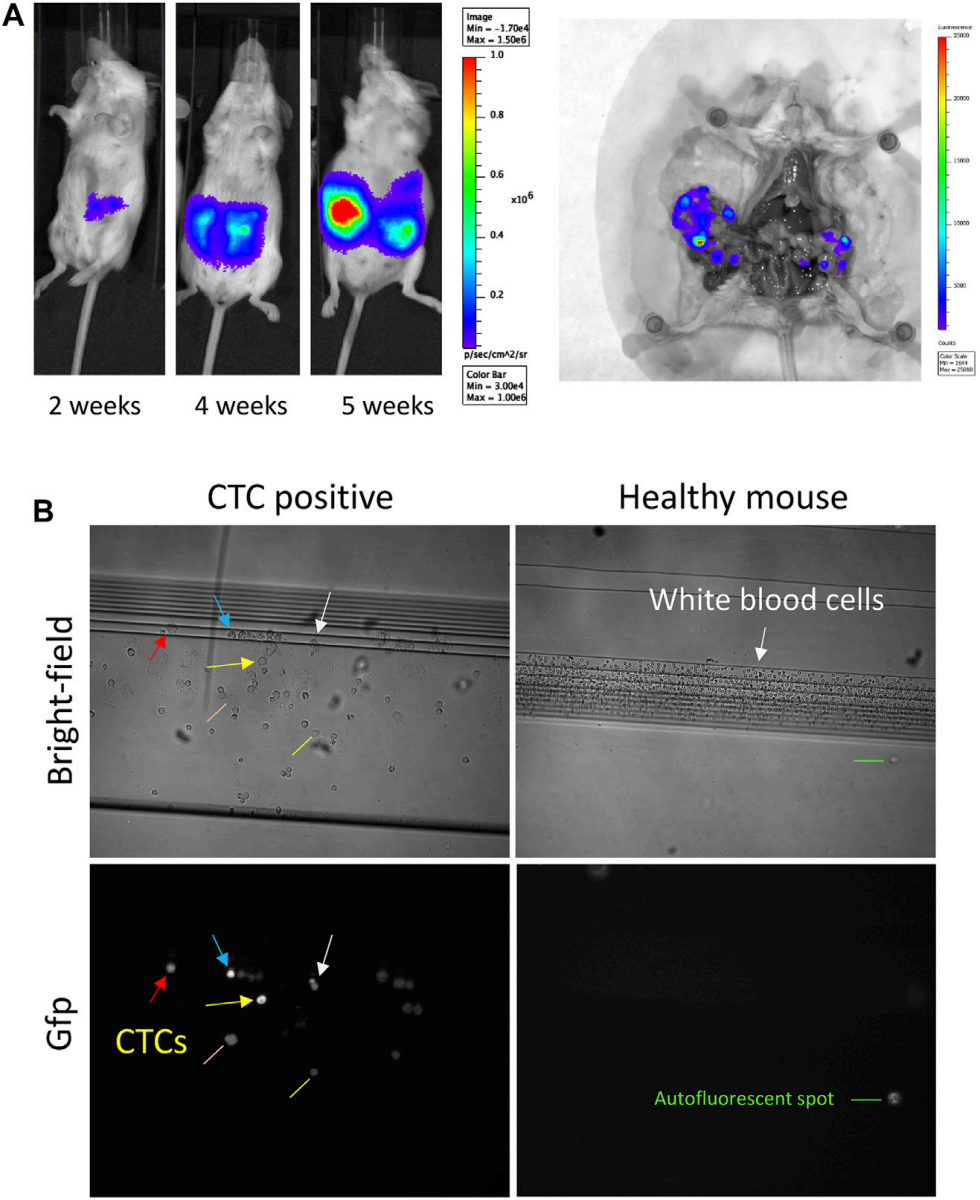
CT26-Luc-Gfp allograft growth in Balb/c mice. **(A)** *In vivo* and *ex vivo* bioluminescence imaging at the indicated times and at sacrifice. **(B)** Bright-field (upper panels) and Gfp fluorescence (lower panels) microscopy of the cells retained in the Parsortix system cassette, from a CTC-positive and a healthy mouse. Arrows of the same color indicate the same cell in the upper and lower panel. Yellow and orange lines indicate cells with lower fluorescence intensity, possibly necrotic. Notice the absence of GFP fluorescence in the healthy mouse cassette, although numerous cells can be seen in brightfield. Green line indicates autofluorescence spot. Although in the image it might appear similar to a cell, it is fluorescent in all channels, thus it can be easily distinguished.

Balb/c mice inoculated with CT26 cells rapidly produce tumors in all the length of the large intestine, even though cells were injected in the cecum wall (not shown). This could be due to cell shedding in the peritoneal cavity and colonizing the rest of the intestinal tube, or due to metastasis through the blood stream. To test the second hypothesis, we decided to search for CTCs derived from these tumors in the blood of the mice. Using a fluorescent and bioluminescent derivative of the cell line, CT26-Luc-Gfp, we inoculated mice orthotopically and monitored tumor growth by *in vivo* imaging (Figure 3A). When mice showed clear sign of metastatic tumor growth (bioluminescent signal expanding in the entire peritoneal cavity), they were sacrificed, and tumor growth and metastasis were confirmed (Figure 3A). At sacrifice, the blood of the mice was extracted and separated in two, for Ficoll gradient nucleated cell isolation and for Parsortix system CTC isolation. We were able to detect the CTCs in the blood of the tumor-bearing mice using fluorescent microscopy, searching for Gfp-positive cells (Figure 3B). On the other hand, we extracted RNA from the mentioned samples to compare the expression levels of our genes of interest in solid tissue, buffy coat, and Parsortix system cassette.

### Gene Expression Varies due to the Presence of CTCs

**FIGURE 4:**
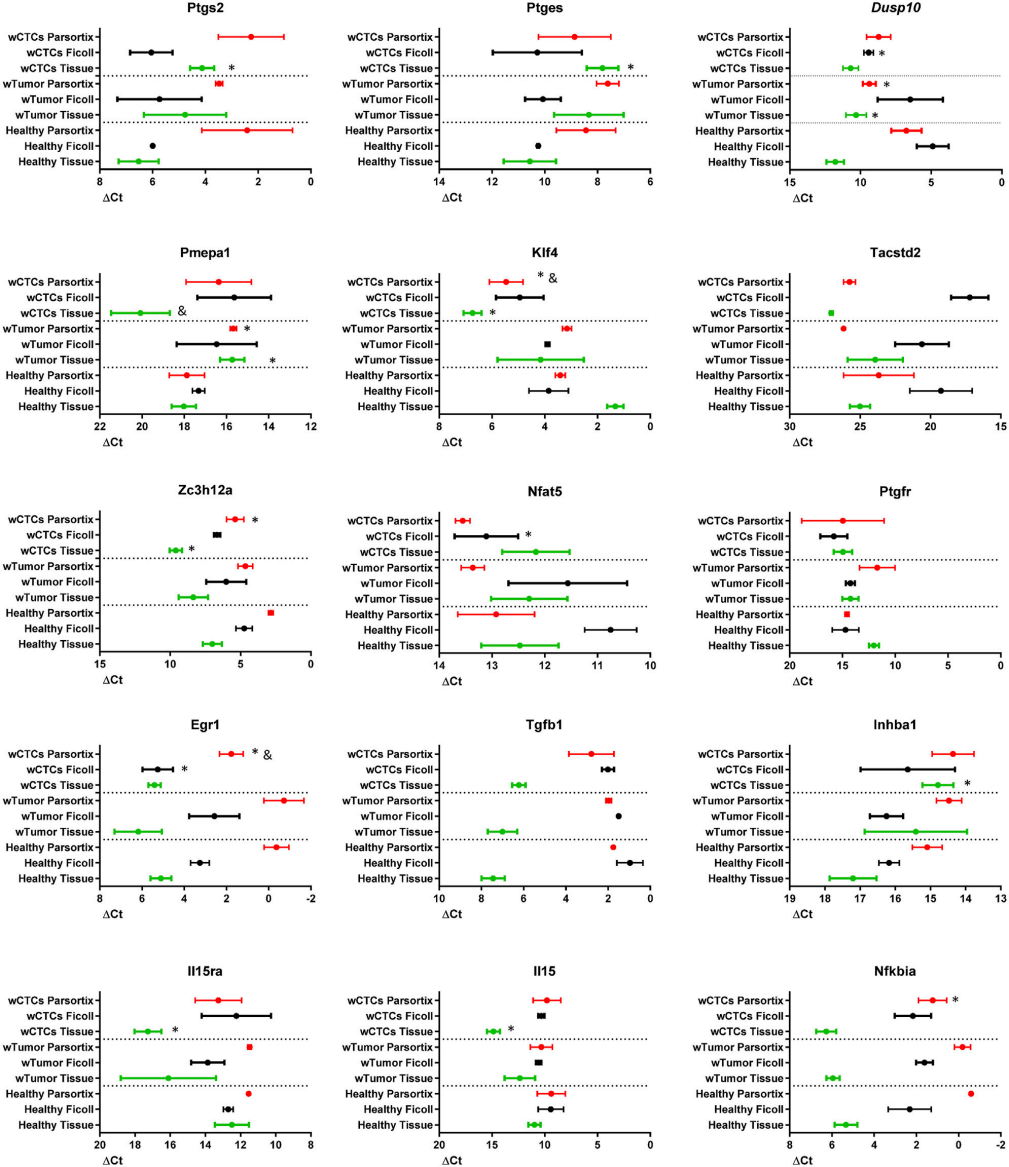
mRNA levels of the indicated genes in solid tissue (green, normal colon or tumor), Ficoll isolate (black), and Parsortix isolate (red) in healthy (*Healthy*), tumor-bearing (*wTumor*), and tumor-bearing with detectable CTCs (*wCTCs*) mice, as estimated by RT-qPCR. ΔCt values are shown, with SEM, plotted on an inversed axis, to facilitate interpretation: left = high ΔCt = low mRNA levels; right = low ΔCt = high mRNA levels. Wilcoxon test was performed for statistical significance. *: p < 0.05 when compared to *healthy* of the same extraction method. &: p < 0.05 when compared to *wTumor* of the same extraction method.

As already shown in Figure 2, the expression of the COX2-effector genes is different between normal colon and colon tumors. We sought to explore if the presence of tumors or tumors releasing CTCs would change the expression pattern of these genes in a detectable way, in solid tissue, in the blood or in the Parsortix system-isolated cell population. Thus, we compared, as shown in Figure 4, the mRNA levels of each gene in solid tissue (green), Ficoll isolate (black), and Parsortix isolate (red) in healthy (*Healthy*), tumor-bearing (*wTumor*) and tumor-bearing with detectable CTCs (*wCTCs*) mice. As expected, the levels of each gene vary between sample/isolation types in each mouse. On the other hand, the tumor presence altered gene expression in a similar way to Figure 2, although it is interesting to note that the *Pmepa1* and *Klf4* expression levels were remarkably lower in tumors with detectable CTCs. In a similar way, regarding Ficoll isolates, *Nfat5* and *Egr1* levels were significantly lower in CTCs bearing samples while the *Dusp10* levels were higher. Finally, *Klf4* and *Egr1* levels were lower in the CTCs containing Parsortix-isolated samples, while *Dusp10* and *Pmepa1* higher levels in these samples could indicate tumor presence. These results suggest that in each isolation type, mRNA level changes can be found that may indicate the presence of tumors or tumors releasing CTCs.

**FIGURE 5:**
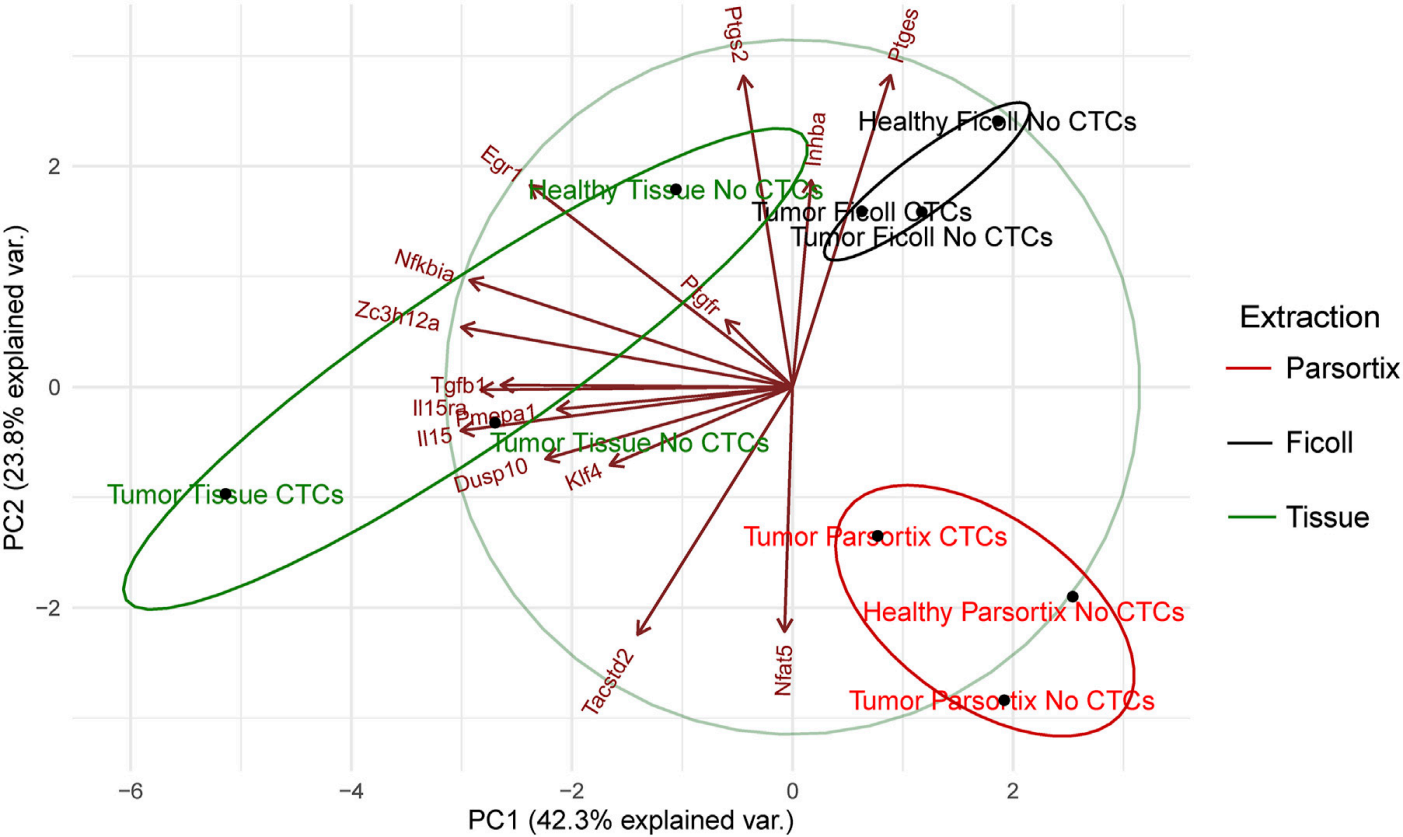
Principal component analysis of the gene expression (ΔCt) data shown in Figure 4, represented as a biplot. Sample principal component score centroids (dots) and loading of variables (vectors) are plotted. Confidence ellipses group samples by extraction type.

To further investigate this, principal component analysis (PCA), taking into account the gene expression levels (ΔCt) in the different “health conditions” and isolation method, was performed. As expected, samples are grouped by extraction method, although in the case of solid tissue, distances are greater than in the other two (Figure 5). In all extraction methods, the CTC-positive groups have lower values for PC1 (influenced mainly by *Egr1*, *Nfkbia*, *Zc3h12a*, *Tgfb1*, *Il15ra*, *Il15*, *Pmepa1*, *Dusp10*, *and Klf4*, as compared with the other two, which by itself would be enough to separate the three health states in solid tissue. PC2 (driven by *Ptgs2*, *Ptges*, *Inhba*, *Tacstd2*, and *Nfat5*) could help distinguish between tumor and healthy, although in the Parsortix samples, healthy falls between the two tumor conditions on the PC1 axis. This confirmed that this group of genes could serve to distinguish between healthy, tumor-bearing, and tumor plus CTC-bearing mice.

## Discussion

COX2 expression and activity has been has been long considered to play a very important role in colon tumorigenesis, cancer progression, and metastasis (Kawai et al., 2002; Cathcart et al., 2011), and its inhibition has been shown to have both great preventive and treatment value (Rothwell et al., 2011; Rothwell et al., 2021). Thus, it is logical to expect a possible role for COX2 in tumor dissemination in the form of CTCs. In fact, other authors have shown a certain association of COX2 expression in CTCs and colon cancer metastasis (Cai et al., 2019). We sought to investigate the mRNA levels of Ptgs2 in our mouse model of colon cancer, CT26 cells-Balb/c mice, as well as those of the group of COX2 effector genes we identified in human colon cancer. First, we confirmed that COX2 activity can change the expression of some of the genes we have identified as COX2-target or effector genes in human cancer (Stamatakis et al., 2015; Jiménez-Martínez et al., 2019; Jiménez-Segovia et al., 2019; Hidalgo-Estévez et al., 2020), namely, *Ptge2*, *Ptges*, *Dusp10*, *Pmepa1*, *Inhba*, *Il15*, and *Nfkbia.* There was also a tendency for *Klf4*, *Tacstd2*, *Nfat5*, *Tgfb1*, and *Il15ra* to be upregulated with COX2 overexpression in CT26 cells, as it happened in HT29 human cells, but without reaching statistical significance. These results indicate that the regulation of these genes is similar in both species, regarding COX2 activity. Cell line-specific effects that could explain the differences cannot be discarded. The regulation of these genes by COX2 was also confirmed when analyzing gene expression in mouse allografts of the CT26 cells. *Ptgfr* levels were lower, both in culture as in tumors, agreeing with the human tumor data (Hidalgo-Estévez et al., 2020) and the notion that, during cancer development and progression, the arachidonate/prostanoid pathway is tuned towards PGE_2_ production and detection, downregulating synthases and receptors of PGF_2α_ and PGD_2_ (Cebola et al., 2015). It is important to note that our mouse allograft model is immunocompetent, simulating better the situation in patients. Immune infiltration, interaction with the tumor cells, and selection pressure may be the responsible for the differences found in the expression of *Klf4*, *Tacstd2*, *Zc3h12a*, *Il15*, *Il15ra*, and *Nfkbia.* Since the implications of the regulation of the COX2-effector genes have been discussed elsewhere, we focus on the gene expression differences when we group individuals for CTCs existence.

The CT26-Luc-GFP–Balb/c singeneic cancer model allowed us to study tumor development and its interaction with the immune system. Thanks to the Parsortix System, we were able to detect CTCs specifically due to Gfp expression by these cells. Thus, when we focused to compare the mRNA levels of each gene in the different sample types, solid tissue, buffy coat, and Parsortix cassette, we were able to group animals not only in healthy and tumor-bearing groups but also for the presence of CTCs. This allowed us to observe an interesting phenomenon regarding Pmepa1 mRNA levels. Although they were higher in tumor tissue than in normal colon, this was not so in tumors of mice with CTCs detected, where Pmepa1 mRNA levels were much lower than in healthy colon. PMEPA1 has been found to be highly expressed in normal colon and most colorectal adenocarcinomas and metastases (Brunschwig et al., 2003; Zhang et al., 2019), but it would be interesting to compare tumors that actively disperse CTCs with others that do not. We have show that PMEPA1 increases cell proliferation while it induces E-cadherin expression in ovarian tumor cells (Jiménez-Segovia et al., 2019). While these cells are able to survive and proliferate better, they are less invasive. If this also happens in colon cancer cells, they would have to reduce PMEPA1 levels before invading blood vessels and releasing CTCs. Another possible explanation could be the effect of the tumor microenvironment which can both contribute to gene expression and shedding of CTCs. The combination of easily detectable CTCs (e.g., expressing Gfp) with the Parsortix system could greatly facilitate this kind of study, making it possible to identify the events or characteristics of tumors of the same origin (e.g., CT26-Gfp cells) to produce CTCs or not.

At the liquid biopsy level, the Egr1 and Klf4 mRNA level reduction in the Parsortix samples could be a good marker for the presence of CTCs. This reduction was also observed in the Ficoll gradient samples, indicating that the level change is probably due to a more generalized change in the expression of these genes in peripheral blood lymphocytes, the percentage of CTCs not being responsible, as it is negligible in the case of the buffy coat. Further studies comparing broad gene expression between healthy, tumor-bearing, and CTC-positive tumor-bearing individuals’ liquid biopsy samples will be able to provide accurate biomarkers for CTC presence or even for cancer presence in general, especially single-cell RNAseq. This is particularly supported by the fact that by just analyzing a small group of genes, we were able to identify two genes of which expression levels could indicate the presence or absence of CTCs. Moreover, taking into account the mRNA levels of the 15 selected genes, we could perform a principal component analysis that clearly separated the healthy, the tumor positive–CTC-negative, and the tumor positive–CTC-positive mice. Single-cell RNAseq studies on Parsortix-isolated cells could increase the robustness of this liquid biopsy method as a diagnostic tool for colorectal cancer.

## Data Availability

The original contributions presented in the study are publicly available. This data can be found here: https://xenabrowser.net/datapages/?cohort=TCGA%20Colon%20Cancer%20(COAD)&removeHub= https:%3A%2F%2Fxena.treehouse.gi.ucsc.edu%3A443.
